# Chemogenetic Excitation of Ventromedial Hypothalamic Steroidogenic Factor 1 (SF1) Neurons Increases Muscle Thermogenesis in Mice

**DOI:** 10.3390/biom14070821

**Published:** 2024-07-09

**Authors:** Christina A. Watts, Jordan Smith, Roman Giacomino, Dinah Walter, Guensu Jang, Aalia Malik, Nicholas Harvey, Colleen M. Novak

**Affiliations:** 1 School of Biomedical Sciences, Kent State University, Kent, OH 44242, USA; cnovak13@kent.edu; 2 College of Public Health, Kent State University, Kent, OH 44242, USA; 3 Department of Biological Sciences, Kent State University, Kent, OH 44242, USA; 4 Brain Health Research Institute, Kent State University, Kent, OH 44242, USA

**Keywords:** muscle thermogenesis, chemogenetics, DREADD, predator odor, SF1, energy balance, obesity

## Abstract

Allostatic adaptations to a perceived threat are crucial for survival and may tap into mechanisms serving the homeostatic control of energy balance. We previously established that exposure to predator odor (PO) in rats significantly increases skeletal muscle thermogenesis and energy expenditure (EE). Evidence highlights steroidogenic factor 1 (SF1) cells within the central and dorsomedial ventromedial hypothalamus (c/dmVMH) as a modulator of both energy homeostasis and defensive behavior. However, the brain mechanism driving elevated EE and muscle thermogenesis during PO exposure has yet to be elucidated. To assess the ability of SF1 neurons of the c/dmVMH to induce muscle thermogenesis, we used the combined technology of chemogenetics, transgenic mice, temperature transponders, and indirect calorimetry. Here, we evaluate EE and muscle thermogenesis in SF1-Cre mice exposed to PO (ferret odor) compared to transgenic and viral controls. We detected significant increases in muscle temperature, EE, and oxygen consumption following the chemogenetic stimulation of SF1 cells. However, there were no detectable changes in muscle temperature in response to PO in either the presence or absence of chemogenetic stimulation. While the specific role of the VMH SF1 cells in PO-induced thermogenesis remains uncertain, these data establish a supporting role for SF1 neurons in the induction of muscle thermogenesis and EE similar to what is seen after predator threats.

## 1. Introduction

Physiological demands require homeostatic regulation, affecting multiple systems, including energy balance [1,2]. Shifts within this critical relationship between energy intake and expenditure have been identified as a primary source for many diseases, including obesity and diabetes [3,4,5,6]. However, allostatic theory suggests that the purposeful induction of homeostatic imbalances to acute challenges may be necessary to support survival mechanisms to actual or perceived stressors, including predator threats [1,7,8]. Predation is a natural component of most ecosystems, leading to evolved innate physiological responses by prey to survive such threats [9,10]. The optimal adaptation of these responses requires precise control over both the initiation and termination of these short-term energy loads, thereby maintaining both survival and the long-term stability of the internal environment [11]. Though the ability of predatory threats to alter the balance between energy intake and energy expenditure (EE) is well established [12], the underlying mechanisms that drive allostatic adaptations in EE have yet to be elucidated.

Predators provoke behavioral responses, such as freezing and escaping to shelter, along with physiological responses of the endocrine and autonomic systems [13,14,15,16]. Less is known regarding the importance of skeletal muscle in the response to predator threats. We have previously established that mice and rats produce robust muscle thermogenic and metabolic responses to the odor of a ferret [17], a natural predator [18]. Moreover, mounting evidence supports a role for similar or overlapping hypothalamic pathways in the regulation of both peripheral energy homeostasis and the response to a predator threat [14,19,20,21]. We therefore investigated the potential for a neural regulator of this allostatic response.

The ventromedial hypothalamus (VMH) in particular is a primary candidate to regulate the thermogenic and metabolic response to antipredator behaviors, as it has been identified as a mediator of predator threats and defensive behaviors [14,15,16,22,23,24,25,26]. The VMH is understood to be sub-sectioned into three functional regions—ventrolateral (vlVMH), central (cVMH), and dorsomedial (dmVMH) [14]. Categorical assessment of these regions has identified the central and dorsal combined regions of the VMH (c/dmVMH) to be primarily responsible for defensive behaviors specifically to predator threats [27], while the vlVMH has been associated with behavioral responses particular to conspecifics [15,28]. Straddling the central and dorsomedial regions lies the steroidogenic factor 1 (SF1; VMH^SF1^) neuronal subpopulation. With the experimental modulation of VMH^SF1^ neurons, mice show increased safety-seeking behavior [13,22,29], freezing followed by activity bursts [14,27], and escape jumping [22]. Parallel methodology demonstrates that the increased neural activity of SF1 neurons also influences metabolic changes, including the suppression of food intake [30] and increased energy expenditure [19,31], glucose utilization [19,32,33], and insulin sensitivity [34]. These effects extend to the peripheral tissues (e.g., skeletal muscle and liver), influencing glucose uptake [35] and insulin sensitivity [36]. Thus, we hypothesized that VMH^SF1^ neurons play a critical role in regulating energy balance during predator odor exposure. Using a chemogenetic approach with the combined technology of temperature transponders and indirect calorimetry, here, we present the findings from SF1-Cre mice exposed to predator odor (PO, e.g., ferret odor) compared to transgenic and viral controls that implicate VMH^SF1^ cells in metabolic and thermogenic outcomes akin to those stimulated by predator threats.

## 2. Materials and Methods

### 2.1. Animals

Animals and all procedures were approved by the Kent State University Animal Care and Use Committee. Adult SF1-Cre mice (FVB-Tg(Nr5a1-cre)7Lowl/J, Jackson Laboratory), both hemizygous (Cre+) mice expressing Cre recombinase in SF1 cells and wild-type noncarriers (WT), were obtained and then maintained via breeding at the Kent State University Department of Biological Sciences vivarium. All animals were genotyped via polymerase chain reaction (PCR) to identify whether they were WT or Cre+. Cre+ (N = 30, male = 20; female = 10), and noncarrier WT mice (N = 8, male = 4; female = 4) of adult age (>6 months) were utilized in this study. Each mouse was housed with same-sex littermates after weaning until surgery. After surgery, mice were individually housed for the remainder of the study. Mice were maintained on a 12/12 h light/dark cycle and received standard chow (5P00 ProLab RMH 3000, LabDiet, St. Louis, MO, USA) and water ad libitum. To minimize thermogenic influences of the dark cycle, all studies were conducted in the middle of the light phase approximately two to three hours after lights-on [31,37].

### 2.2. DREADD Constructs

**Figure 1:**
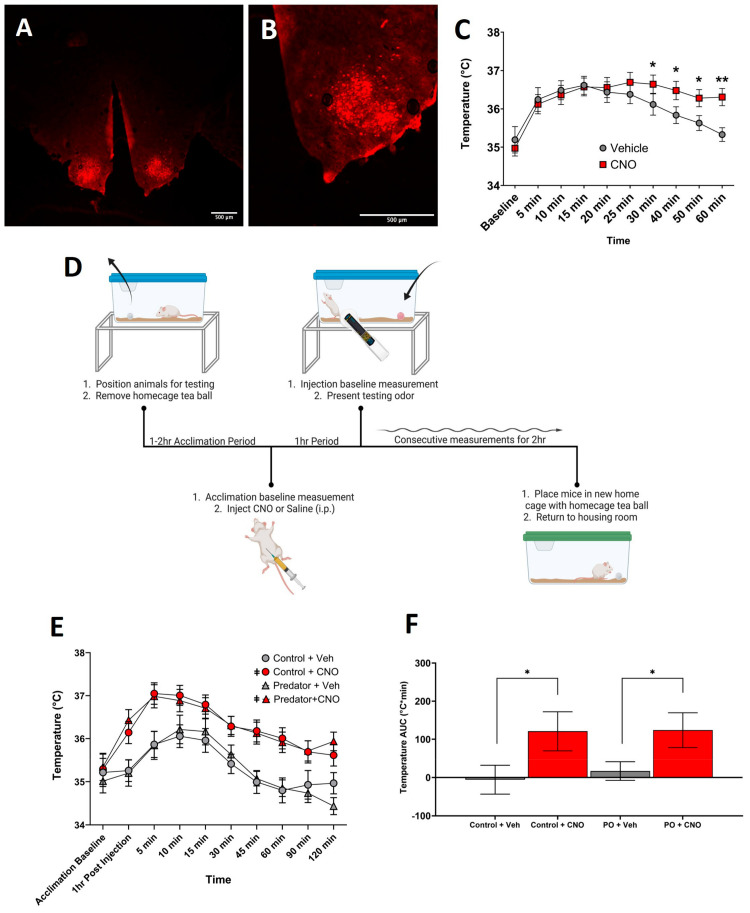
The chemogenetic activation of ventromedial hypothalamic (VMH) steroidogenic factor 1 (SF1) neurons significantly elevated muscle temperature compared to the vehicle condition. (**A**,**B**) The representative fluorescent image of mCherry expression in the VMH following the transduction of the control construct in an SF1-Cre+ mouse. (**C**) Gastrocnemius muscle temperature following CNO or vehicle injection (1 mg/kg, i.p.). Mice were measured after acclimating to the testing room prior to injection, at (Baseline), and consecutively for one hour. (**D**) The workflow of the home cage testing protocol. (**E**,**F**). The gastrocnemius muscle temperature of SF1-Cre+ mice transduced pAAV-hSyn-DIO-hM3D(Gq)-mCherry (AAV8) following CNO or the vehicle (1 mg/kg, i.p.). Thermogenesis was measured in all mice in each condition. N = 14, ǂ main effect of dose, * *p* < 0.05, ** *p* < 0.01, error bars represent ±SEM. PO, predator odor; Control, control odor; CNO, clozapine-N-oxide; Veh, vehicle (sterile saline).

Two DREADD constructs were utilized in SF1-Cre+ mice. To examine the influence of VMH^SF1^ neurons’ increased excitability, (1) pAAV-hSyn-DIO-hM3D(Gq)-mCherry (AAV8) (Addgene, Watertown, MA, USA) was used. As a control for Cre+ response to the excitatory DREADD vector, WT mice were also stereotaxically injected with this construct (Figure S1). For comparison, a mCherry control adeno-associated virus (AAV) of the same serotype was also used: (2) pAAV-hSyn-DIO-mCherry (AAV8) (Addgene, Watertown, MA, USA). A representative image of positive mCherry expression within the VMH is displayed in Figure 1A,B. Clozapine-N-oxide (CNO), dissolved in sterile saline, was used as the DREADD ligand activator. CNO was an optimal choice for this study, as it is known to cross the blood–brain barrier [38,39]. Of the range of doses of CNO tested prior to this study [40], we found that a standard dose of 1.0 mg/kg was sufficient to impact thermogenesis while minimizing potentials for off-target effects [41].

### 2.3. Stereotaxic (AAV) and Transponder Implantation Surgery

Mice were anesthetized using 4–5% isoflurane and maintained throughout surgery at 1–2.5% isoflurane. Mice were provided two forms of analgesia, 0.1 mg/kg of buprenorphine and 5 mg/kg of ketoprofen. After preparation of the mouse and surgical site, the following coordinates were used for injection into the c/dmVMH: +/−0.4 mm lateral to bregma, −1.3 mm posterior to bregma, and −5.3 mm ventral to dura. Bilateral 200 nL injections of AAVs (hM3Dq or control constructs) were administered using a NeuroS Hamilton syringe over a 5 min period. The withdrawal of the syringe followed a 5 min additional delay. During this period, IPTT-300 transponders (BioMedic Data Systems, Seaford, DE, USA) were implanted adjacent to the right hind-limb gastrocnemius muscles (between medial and lateral groups). IPTT-300 transponders provide a mode of identification and distant temperature measurement of the gastrocnemius muscle tissue. Each mouse was placed in a new home cage after surgery. Home cages included a ‘home-cage tea ball’, secondary enrichment mimicking the testing odor stimulus, as described in Watts et al. [42]. After surgery, mice were administered 0.05 mg/kg buprenorphine 6 h after surgery and the morning after surgery. An amount of 5 mg/kg ketoprofen was provided in the afternoon the day after surgery and the following day; post-op care, mouse weight, and temperatures were logged daily. Monitoring continued until the mice gained or maintained weight for a minimum of 2 consecutive days.

All mice were given 3 weeks for recovery and to allow for the sufficient expression of DREADD. Beginning the final week of recovery, mice were habituated over four trials to the experimental context [42]. Each habituation consisted of animals transitioning to assigned locations within the testing facility, removal of home-cage tea ball, one hour acclimation to the testing room, return of the home-cage tea ball to simulate the introduction of a novel odor, and consecutive measurements for one hour using a transponder reader (DAS-700R, 7007S, 8027 BioMedic Data Systems, Seaford, DE, USA). Following each habituation session, animals were returned to their housing room.

### 2.4. CNO Effect Muscle Temperature

To examine the independent influence of CNO directly after injection, mice habituated for temperature testing were injected with either a saline vehicle or CNO (1.0 mg/kg, i.p.). All mice were tested in a randomized order of experimental conditions. Mice were transferred to the testing facility and given one to two hours to acclimate. After acclimation, baseline temperature was measured using a transponder reader. Each animal received an injection, and then, measurements occurred consecutively for one hour.

### 2.5. Home-Cage PO-Induced Muscle Temperature

All mice experienced each condition with a minimum of 48 h between experimental exposures to prevent carryover effects. Temperature measurements were obtained after one to two hours of acclimation and one hour after an injection of either CNO or vehicle (sterile saline). Mice were then presented with control or predator odor (PO) via tea ball. Control-odor towels were prepared using a clean 2” × 2” towel enclosed in a stainless-steel mesh tea ball. PO was provided via similar-sized cuts of towels that were used as bedding for a ferret over a two-week period (Marshall BioResources, North Rose, NY, USA). The tea ball gives a familiar barrier between the mice and the towel, minimizing physical activity that can occur in response to interactions with the towel material [42]. Once the odor was presented, animals were measured consecutively for two hours (Figure 1D).

### 2.6. Treadmill Walking and PO-Induced Muscle Temperature

**Figure 2:**
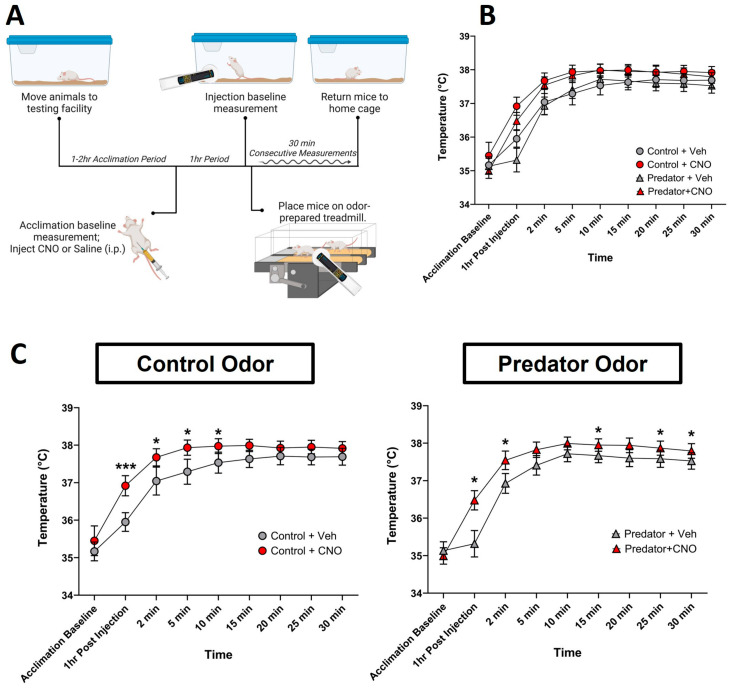
The chemogenetic activation of ventromedial hypothalamic (VMH) steroidogenic factor 1 (SF1) neurons significantly elevated muscle temperature during prescribed treadmill walking. (**A**) The workflow of controlled activity temperature testing via treadmill walking at an incline of 0° and a walking speed of 5.2 m/min. (**B**) Gastrocnemius muscle temperature following CNO or vehicle injection (1 mg/kg, i.p.) in SF1-Cre+ mice transduced with pAAV-hSyn-DIO-hM3D(Gq)-mCherry (AAV8), excitatory DREADD. (**C**) The *t*-test analysis by odor comparing CNO to vehicle injections. N = 12, * *p* < 0.05, *** *p* < 0.001, error bars represent ±SEM. CNO, clozapine-N-oxide; Veh, vehicle (sterile saline).

Treadmills were set to a walking speed of 5.2 m/min, with shockers used as motivation for continual movement. All mice were habituated to treadmill walking before testing by walking once for 15 min. On the day of testing, baseline temperatures were measured prior to placement onto the treadmill. Mice were then administered either CNO (1.0 mg/kg, i.p) or saline vehicle. One hour after injection, temperatures were measured to collect a post-injection baseline. Treadmills were then prepared with control or PO in accordance with the experimental condition. Mice were then placed on pre-assigned treadmills; treadmills and shockers were then turned on, and consecutive measurements were collected over 30 min (Figure 2A).

### 2.7. Indirect Calorimetry

**Figure 3:**
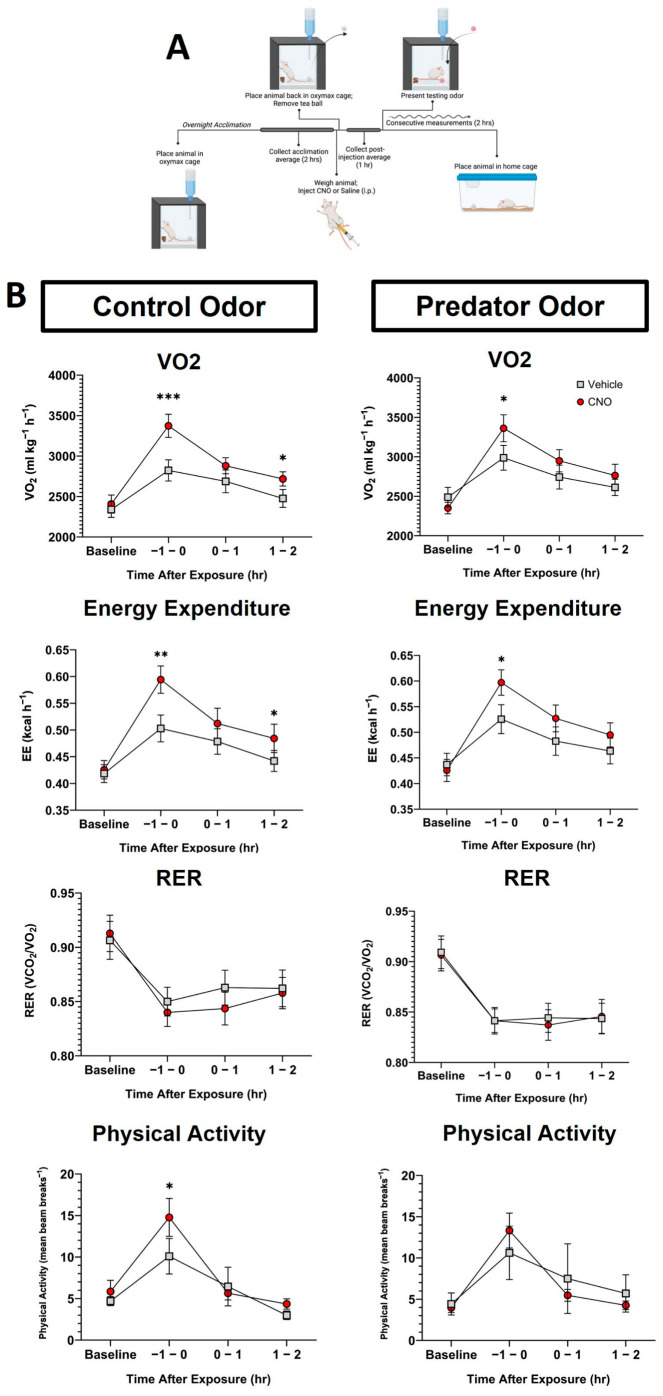
The chemogenetic activation of ventromedial hypothalamic (VMH) steroidogenic factor 1 (SF1) neurons elevated energy expenditure (EE) in mice even in the absence of altered physical activity. (**A**) The workflow of indirect calorimetry following a clozapine-N-oxide (CNO) or vehicle (i.e., saline) injection and the presentation of the predator or control odor. (**B**) The oxygen consumption (VO_2_), energy expenditure (EE), respiratory exchange ratio (RER; VCO_2_/VO_2_), and physical activity of SF1-Cre+ mice transduced with pAAV-hSyn-DIO-hM3D(Gq)-mCherry (AAV8) following CNO or vehicle (1 mg/kg, i.p.) and presented with [left] control odor and [right] predator odor. All mice were tested in each condition. N = 14, * *p* < 0.05, ** *p* < 0.01, *** *p* < 0.001, error bars represent ±SEM. CNO, clozapine-N-oxide; Vehicle, sterile saline.

Animals were placed in an Oxymax FAST system with infrared activity monitors (Columbus Instruments, Columbus, OH, USA). Comprehensive lab animal monitoring system (CLAMS) Oxymax software (Columbus Instruments, Columbus, OH, USA) was used to assess data acquisition. Mice were moved to the chamber and placed into calorimetry-specific cages along with their home-cage tea ball two to three days prior to testing to acclimate to the new housing space. After acclimation to the space, mice within calorimetry cages were weighed and returned to cages, which were connected to the instrument overnight. Baseline acclimation measurements were calculated over two hours on the day of testing. Following acclimation assessment, home-cage tea balls were removed, and mice were re-weighed, administered an injection of either CNO (1.0 mg/kg, i.p.) or saline vehicle, and returned to calorimetry cages. Baseline measurements were collected one hour after injection, then animals were presented with an odor—either control or PO—via tea ball [42]. Consecutive measurements were taken over a two-hour period. Following the experiment, animals were removed from calorimetry cages and returned to their home cage during calorimetry-cage cleaning (Figure 3A). Mice are then returned to clean calorimetry cages. All animals lived within calorimetry appropriate cages throughout the duration of the experimentation in each condition, approximately seven days.

### 2.8. Indirect Calorimetry with Treadmill Walking

Mice were habituated to walking at 5.2 m/min over a 15 min period prior to testing day on treadmills connected to the Oxymax FAST system. On the day of testing, mice were given an injection of either saline vehicle or CNO (1.0 mg/kg, i.p) and then moved to odor-prepared treadmills with either control or PO. Mice were then placed on pre-assigned treadmills; treadmills and shockers were then turned on, and consecutive measurements were collected over 30 min while mice walked on the treadmill at constant speed.

### 2.9. EchoMRI

Body composition analysis of each animal was completed after indirect calorimetry assessment using an EchoMRI-700 (Echo Medical Systems, Houston, TX, USA) system. Each animal was weighed and then placed inside the instrument to assess fat mass and lean mass.

### 2.10. Histology

Mice were transcardially perfused with phosphate buffered saline (PBS) followed by 4% paraformaldehyde in phosphate buffer. Brains were post-fixed for 24 h and then saturated in 30% sucrose. Using a Leica cryostat, 30 μm coronal sections were obtained and then mounted onto a positively charged slide and dried at room temperature. Counterstaining was completed with mounting media containing DAPI (ProLong™ Gold Antifade Mountant with DAPI, Thermofisher, Waltham, MA, USA). Coverslips were then placed and sealed with clear nail polish. To visualize mCherry fluorescence in both the DREADD and control vector, an Olympus FLUOVIEW FV3000 confocal laser scanning microscope (Olympus, Breinigsville, PA, USA) was used. Laser excitation was set to 594 nm with an emission of 608–683 for mCherry.

### 2.11. Statistics

Photobleaching of brain sections from multiple mice prevented complete histological verification; thus, we opted to use a functional physiological verification of successful DREADD expression. Zhang (2020) indicated that successful chemogenetic activation of VMH^SF1^ neurons was followed by a 20% or greater increase in energy expenditure (EE) [30]. Given this limitation and established relationship between EE and VMH^SF1^ neurons, data from SF1-Cre+ mice transduced with an excitatory DREADD in the present study were only included if they met the single criterion of having a 20% increase in EE relative to vehicle condition. A table to describe statistical findings before the implementation of this criterion are displayed in Table S1. Primary analysis of the data was performed using IBM SPSS Statistics version 28 and Prism-GraphPad by Dotmatics version 10.2.2, with averages and standard error of the mean (SEM) calculated using Microsoft Office Excel when necessary. Post-hoc analysis to identify specific interactions found in the primary analysis was performed using Microsoft Office Excel version 16. Figures were created with Prism-GraphPad by Dotmatics version 10.2.2. Three-way repeated measures ANOVA were performed on all data to assess potential interactions between our within-subject variables, dose (CNO or vehicle), odor (PO or control), and time. Area under the curve (AUC) for temperature was calculated using the trapezoidal method described previously [17] and compared dose and odor using IBM SPSS Statistics version 28. Time points changed respective to the specific study: raw temperature, 10 time points; controlled activity raw temperature, 9 time points; indirect calorimetry, 4 time points; controlled activity indirect calorimetry, 7 time points. Each animal underwent all possible within-subject conditions in a counterbalanced randomized order, increasing statistical power.

## 3. Results

### 3.1. Chemogenetic Activation of VMH^SF1^ Neurons Increases Muscle Temperature 

To probe the activation of SF1 neurons following DREADD-induced excitation via CNO, we measured the thermogenic response of skeletal muscle for one hour following injection in a home-cage setting. While i.p. injection of both CNO or the vehicle elicited a marked increase in muscle temperature at the initiation of the test, the chemogenetic activation of VMH^SF1^ cells maintained the elevated muscle temperature for the duration of the testing period (Figure 1C). This was demonstrated in statistical main effects of both time and CNO and a significant interaction of time by CNO. In contrast, SF1-Cre mice infected with a mCherry control vector showed no significant main effect of CNO (Figure S2A). The further analysis of potential sex differences revealed that the muscles of female mice were significantly warmer than males at baseline, prior to injection through 40 min (*p* < 0.05), when injected with the vehicle, saline. In comparison, female mice receiving CNO were significantly warmer than littermate males only at baseline and 5 min (*p* < 0.05 and 0.01, respectively). Thus, the increased activity of VMH^SF1^ cells is associated with increased thermogenic output from the gastrocnemius.

After identifying the effects of VMH^SF1^ neurons independent of stimulus, we then sought to determine if the introduction of predator odor (PO) after the chemogenetic activation of these neurons would increase the thermogenic output. Here, while we found the predicted significant main effects of time and CNO (*p* < 0.0001 and 0.04, respectively) and a significant interaction of time by CNO (*p* < 0.0001) both over time and for temperature AUC, there was not a significant main effect of odor (Figure 1E,F). The ability of CNO to elevate muscle temperature was seen exclusively in animals treated with an excitatory DREADD (pAAV-hSyn-DIO-hM3D(Gq)-mCherry (AAV8)) in comparison to mice that received the control virus (Figure S2B). A sex analysis within each odor paradigm revealed sporadic differences between males and females. Here, the muscles of females were warmer than males at 5 min and again from 15 to 45 min (*p* < 0.05) in vehicle conditions when exposed to predator odor. With CNO, females revealed an increased thermogenic output compared to males at baseline, 15 min, and 30 min (*p* < 0.05). No significant differences between sex were observed in control-odor conditions. These results confirm that while the excitation of VMH^SF1^ neurons was able to mimic an increase in muscle temperature previously observed in response to PO exposure [17], the dual stimulation of excitation and PO did not result in the further elevation of muscle temperature.

Recognizing that the free-range mobility of the mice in the home-cage setting could influence the muscle temperature, we equalized this variable using a similar testing paradigm in a controlled activity setting (i.e., treadmill walking). We report significant main effects of time spent walking on the treadmill (*p* < 0.0001) and CNO (*p* = 0.02) and significant interactions of time by CNO (*p* < 0.0001) and time by odor (*p* = 0.0009) (Figure 2). The sex analysis showed incidental differences not relevant to the experimental question. This included single points of increased temperature between acclimation baseline and the 5 min measurement in both control and PO conditions (*p* < 0.05). In females that received CNO during the PO condition, a significant increase in temperature compared to males was observed in the final two measurements of this test (*p* < 0.05). The ability of CNO-induced DREADD activation to increase muscle temperature during treadmill walking reinforces the relevance of VMH^SF1^ cells to muscle thermogenesis, even when potential differences in muscle contraction are equalized through controlled activity.

### 3.2. Chemogenetic Activation of VMH^SF1^ Neurons Increases Energy Expenditure

To determine the effect of the chemogenetic activation of VMH^SF1^ neurons on metabolic responses during predator odor exposure, we assessed mice transduced with an excitatory or control virus in an indirect calorimeter (Oxymax). As predicted, the i.p. injection of CNO resulted in a significant main effect of time (*p* < 0.0001) and CNO (*p* = 0.02) as well as an interaction of time by CNO (*p* = 0.002), reflecting increases in both energy expenditure (EE) and oxygen consumption (VO_2_) with CNO (Figure 3B). However, statistically significant changes to EE and VO_2_ were specific to the first hour after CNO was injected and before the presentation of the odor stimulus. In contrast to the CNO-dependent changes during activity, respiratory exchange ratio (RER) showed only a significant main effect of time (*p* < 0.0001), with RER decreasing during the light (inactive) phase of the cycle (Figure 3B). The analysis of sex differences showed significant differences only in VO_2_. Here, females had a significantly higher VO_2_ at baseline compared to males before exposure to odor stimulus or injection (*p* < 0.05). Periodic differences were also seen in vehicle conditions at −1–0 h, 0–1 h, and 1–2 h (*p* < 0.01, 0.05, 0.001, respectively) in control conditions, where females had significantly higher VO_2_ than males. In the PO–vehicle paradigm, differences were observed only at the 0–1 h (*p* < 0.05) time point where females had higher VO_2_; this was likely due to their smaller body size.

**Figure 4:**
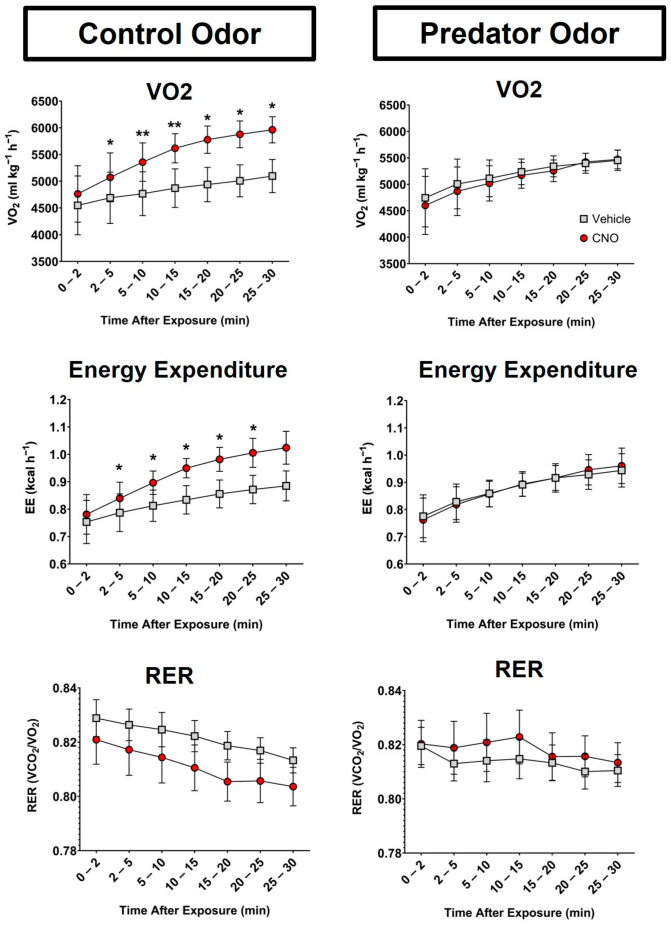
The chemogenetic activation of ventromedial hypothalamic (VMH) steroidogenic factor 1 (SF1) neurons during prescribed treadmill walking. Oxygen consumption (VO_2_) [**top**], energy expenditure (EE) [**center**], and the respiratory exchange ratio (RER) [**bottom**] during controlled activity (e.g., treadmill walking) of SF1-Cre+ mice transduced with pAAV-hSyn-DIO-hM3D(Gq)- mCherry (AAV8) following CNO or the vehicle (1 mg/kg, i.p.) and presented with control odor [**left**] and predator odor [**right**]. All mice were tested in each condition. Mice were consecutively measured for 30 min, 1 h following injection. N = 13, * *p* < 0.05, ** *p* < 0.01, error bars represent ±SEM. CNO, clozapine-N-oxide; Vehicle, sterile saline.

To determine the potential contribution of activity EE to the metabolic outcomes, EE was assessed during controlled activity using indirect calorimetry during the prescribed treadmill walking. Similar to the home-cage calorimetry analysis, a main effect of time was observed over the course of the testing period, showing elevated EE (*p* = 0.02) and RER (*p* < 0.0001) with a similar trend in VO_2_ (*p* = 0.059) during treadmill walking (Figure 4). However, interactions between time and CNO were observed for all metabolic parameters: EE (*p* = 0.0036), VO_2_ (*p* = 0.0001), and RER (*p* = 0.02). While paired sample *t*-tests revealed time points where EE and VO_2_ showed significant elevation with CNO-induced VMH^SF1^ cell activation, further investigation did not identify any time points significantly altered in RER. No significant differences were identified between male and female mice.

Mice were then subjected to a body composition analysis after metabolic testing to assess fat and lean mass (Table 1). Unpaired two-tailed *t*-tests were performed on males, females, and combined sex cohorts to assess potential differences between mice that received either control or excitatory constructs. While female mice showed significantly lower lean mass (*p* = 0.033) in those that received an excitatory construct, the assessment of males revealed significantly higher fat mass (*p* = 0.026) and body weight (*p* = 0.027) in those that received the excitatory construct. However, the analysis of the combined cohorts revealed no significant differences in fat (*p* = 0.507) or lean (*p* = 0.914) body composition; no significant differences were observed in body weight (*p* = 0.530). Overall, this shows that the different viral constructs did not significantly alter body composition in ways that may have influenced their metabolic and thermogenic responses examined here.

## 4. Discussion

Given the importance of the VMH^SF1^ cell population in both behavioral responses to predator threats and skeletal muscle metabolic regulation [22,33,43], we hypothesized that this cell group is also important in muscle thermogenic and metabolic outcomes seen in the presence of predator threats. Using the DREADD-induced activation of VMH^SF1^ neurons, we sought to understand if increased neural activity would (1) increase skeletal muscle thermogenesis and (2) increase energy expenditure. Though we did not replicate strong thermogenic and metabolic responses to the presence of PO seen previously [17], this study confirmed that the excitation of VMH^SF1^ neurons increases muscle thermogenesis along with elevating EE and oxygen consumption. Though the VMH has been linked to brown adipose tissue activation and muscle insulin response [44,45,46], to our knowledge, this is the first demonstration of a rapid muscle thermogenic effect derived from the activation of cells in the VMH.

Significant increases in skeletal muscle thermogenesis were observed when VMH^SF1^ neurons were chemogenetically activated; however, elevated muscle temperatures were seen regardless of the presence or absence of PO. This was surprising given that evidence using this and other mouse lines supports the ability of predator—specifically ferret—odor to induce muscle thermogenesis in mice [17,43,47]. Additionally, continued work with this mouse line, in our hands, has replicated the previously published thermogenic response [47]. However, a detailed observation of the data suggests that the stress-induced elevation in muscle temperature seen after mouse handling and i.p. injection overshadowed any thermogenic effects stemming from the later presentation of PO. Moreover, the chemogenetic activation of VMH^SF1^ resulted in significant thermogenesis alone, potentially obscuring thermogenesis stemming from the odor exposure. It should be noted that mice injected with a control virus and all treatments with the saline vehicle also showed no significant thermogenic effect when presented with PO (Figure S2). Similarly, though prior evidence supports the ability of PO exposure to increase EE [17], no PO-induced elevation in EE was detected here (Figure 3B) apart from an interaction in the effects of CNO and PO during treadmill temperature assessment (Figure 2C). Regardless, this dataset demonstrates the ability of this VMH^SF1^ cell population alone to induce skeletal muscle thermogenesis, even in the absence of a predator threat, thus implicating these cells in the modulation of muscle thermogenesis. The ability of VMH^SF1^ cells to mediate PO-induced muscle thermogenesis specifically, however, remains unanswered.

The chemogenetic excitation of VMH^SF1^ induced a significant increase in EE and oxygen consumption during the first hour after injection (Figure 3B). These data suggest that VMH^SF1^ neurons have an immediate and active influence on metabolic rate. This could stem in part from elevated physical activity; however, relatively elevated oxygen consumption and EE were seen even when physical activity was not significantly enhanced (Figure 3B). This suggests an additional contribution of locomotor inefficiency (e.g., lower skeletal muscle work efficiency or higher cost of transport) to the elevated EE seen with VMH^SF1^ activation. This aligns with established evidence where the chemogenetic activation of VMH^SF1^ neurons drives increases in energy expenditure while not influencing physical activity [30,33,35]. Nevertheless, this increase in EE is not sustained throughout the duration of measurement (Figure 3B). These outcomes do not rule out other potential mediators for muscle thermogenesis that are known regulators of metabolic rate fluctuations, such as proopiomelanocortin (POMC) neurons within the arcuate nucleus [3,48,49].

Calorimetry also gives insight into ways in which fat is being utilized as a source of energy. Though previous evidence has suggested that the chemogenetic activation of SF1 cells results in decreased RER, implicating increased fat oxidation [35], no significant changes in RER were seen with the chemogenetic activation of VMH^SF1^ neurons relative to vehicle control conditions (Figure 3B). However, a sharp decrease in RER was observed in the first hour after injection relative to baseline measurement. This response may stem from a potential time-of-day effect, where residual influences of the dark cycle may influence RER. Overall, these SF1 neurons influence energy expenditure without bias in substrate utilization under sedentary conditions in the home-cage setting.

In freely moving rats exposed to PO, increased physical activity has been noted [17]. However, here, we identified no significant influences of either PO or the activation of VMH^SF1^ neurons on physical activity in mice outside of the predictable influence of handling and injection (Figure 3B). These data suggest that the activation of SF1 neurons has minimal influence on locomotor activity. Moreover, the lack of hyperactivity suggests that the metabolic outcomes documented here, including elevated EE, likely do not stem from elevated physical activity alone. Similarly, exposure to PO elevated EE and muscle temperature in rats even when activity was controlled [17], similar to the elevation in EE and thermogenesis seen here in mice with the chemogenetic activation of VMH^SF1^ neurons (Figure 1 and Figure 3). Altogether, given that the ability of VMH^SF1^ neuronal activation to increase thermogenesis and EE without relatively elevated physical activity was confirmed using the additional procedure of manually controlling activity through prescribed treadmill walking (Figure 2 and Figure 4), this implies that the thermogenesis observed with the chemogenetic activation of VMH^SF1^ neurons does not rely on elevated contractile thermogenesis.

Our consistent evidence of increased thermogenic output and energy expenditure in response to VMH^SF1^ chemogenetic activation implicates a role for this subpopulation in the homeostatic control of energy balance, including muscle thermogenesis. Recognizing sex as a biological variable, we included both male and female mice in our studies. Though sporadic differences were identified during the analysis, we found no systematic sex differences in response to chemogenetic SF1 cell activation. This finding follows our previous report that the estrous cycle does not influence PO-induced thermogenesis [17]. One limitation of this study is that injection site locations for many of the animals utilized could not be confirmed. This allows for a potential of missed injections and the inadequate stimulation of our target population. In response to this concern, we utilized the previously published literature to establish a criterion based on EE shifts after chemogenetic excitation [30,35]. Though not standard, this criterion allows our thermogenic results to build upon an established understanding between VMH^SF1^ neurons and EE. To mitigate potential bias introduced by the use of the inclusion criterion, Table S1 details the congruency of the statistical outcomes before the comparison to after the application of the criterion. There was no meaningful alteration in the conclusions after the two mice that did not reach the criterion for inclusion were eliminated from the analyses. Further, while CNO metabolites have the potential to induce off-target effects [50,51,52,53], the dose employed here minimized this risk. More pertinent is the stress response of the mice following IP injection of the CNO, which likely obscured the ability of ferret odor to elevate muscle temperature, as we have shown previously [17]. Moreover, we are unable to conclusively rule out shivering thermogenesis, even in the absence of visual evidence of shivering. Lastly, we acknowledge that the lack of simultaneous measurement of general body temperature or the temperature of brown adipose tissue (BAT) with muscle temperature reduced our ability to put the muscle thermogenesis into a larger physiological context regarding thermogenesis. Due to the spatial limitation of this model, we were unable to place multiple transponders within a single mouse, limiting our ability to one site of measurement. Evidence consistently points to the ability of VMH cell populations, including SF1, to promote BAT thermogenesis [51,52,53,54,55]. Therefore, it is likely that BAT thermogenesis and the accompanying metabolic activity contributes to the elevated EE seen with VMH^SF1^ cell activation here (Figure 3). Conversely, it is also possible that increased muscle thermogenesis adds to the elevated EE seen in conjunction with activation of the VMH and concomitant BAT thermogenesis.

Given mounting evidence that the VMH is a convergence point for olfactory signals [21,56,57], we hypothesized that the odor of a natural predator (e.g., ferret) activates VMH^SF1^ cells through the olfactory bulb and accessory pathways. These signals would then project to downstream brain regions, such as the medial amygdala, VMH, periaqueductal gray (PAG), and then the rostral ventrolateral medulla, resulting in a sympathetic nervous system response and increased skeletal muscle temperature [17]. However, VMH^SF1^ neurons are also known to alter pathways affecting defensive behaviors. Specifically, downstream projections from the VMH to both the PAG and the anterior hypothalamic nucleus (AHN) mediate defensive behaviors but in opposing fashions where PAG elicits immobility and the AHN is responsible for escape or jumping behaviors [13,22,33]. It can be assumed that responses such as escape and jumping would require increased energy output from skeletal muscle, thereby increasing the requirement for energetic resources. Conversely, actions such as immobility may not require the same extent of the mobilization of energy and therefore not increase caloric expenditure. Consequently, it is probable that the general excitation of VMH^SF1^ neurons provokes opposing responses in which neurons projecting to the AHN (e.g., escape behaviors) prime metabolic outflow for the optimization of escape, while neurons projecting to the PAG (e.g., immobility) are responsible for the reallocation of resources amidst anxiety and fear. Likewise, increased energy expenditure may be a necessary mechanism to adapt to suppressed feeding behaviors observed around predators, thus maintaining safety rather than taking risks to increase energy intake [29].

Variations in the behavioral and physiological responses of the VMH may also stem from the heterogeneity of VMH^SF1^ cells, as there are at least five subpopulations with distinct gene expression patterns colocalized with SF1 nuclear receptors, including leptin receptors (LEPR), insulin receptors (IR), brain-derived neurotrophic factor (BDNF), sirtuin-1 (SIRT1), and glucokinase (GK). Each of these subsets have been purported to have individual and, for some, opposing roles in metabolic output [58,59,60,61]. More recently, an additional subpopulation of VMH^SF1^ neurons expressing pituitary adenylate cyclase-activating polypeptide (PACAP) has been investigated for their specific role in the induction of tissue thermogenesis [62]. Thus, it is probable that different VMH^SF1^ subpopulations could have differing metabolic outcomes, including opposing effects on thermogenesis. Future exploration should investigate these markers to specify the subpopulation that may be responsible for thermogenic and metabolic outflow, including responses to PO.

Taken together with prior evidence in the context of behavioral responses to predator threats, the current study supports a role for VMH^SF1^ neurons in the induction of muscle thermogenesis, similar to the muscle thermogenic response to a predator threat. Given the ability of the chemogenetic activation of VMH^SF1^ neurons to elevate both EE and muscle thermogenesis, the role of VMH^SF1^ neurons in muscle thermogenesis merits continued exploration. Understanding the neural pathway exerted in both thermogenic and energy responses is not only critical for the expansion of the allostatic theory, but it also provides insight into ways in which muscle thermogenesis can be used to restore energy balance in human metabolic disorders, such as diabetes and obesity. Collectively, this work has formed a foundation for establishing the neural pathways mediating muscle thermogenesis.

## Figures and Tables

**Table 1:** Body composition did not differ in mice that received SF1-Cre+ hM3Dq and control vector (Mean ± SEM).

	Fat Mass (g)			Lean Mass (g)			Body Weight (g)		
	Male	Female	All	Male	Female	All	Male	Female	All
Control Vector N = 15	5.800 ± 0.688	5.895 ± 3.249	5.831 ± 1.099	14.676 ± 0.880	12.991 ± 0.461	14.114 ± 0.631	35.781 ± 1.643	32.210 ± 1.643	34.591 ± 1.635
Excitatory Vector N = 14	8.926 ± 1.239	2.465 ± 0.333	7.080 ± 1.193	16.771 ± 1.234	9.682 ± 1.165	14.746 ± 1.277	40.811 ± 1.699	26.275 ± 0.764	36.658 ± 2.187

The body composition of mice within the presented tests. SF1 Cre+ mice transduced with an mCherry control vector (**top**) and mice transduced with pAAV-hSyn-DIO-hM3D(Gq)-mCherry (AAV8) (**bottom**). The average fat mass (**left**), lean mass (**center**), and body weight (**right**) are presented by sex and combined mean by vector.

## Data Availability

The raw data supporting the conclusions of this article will be made available by the authors on request.

## Supplementary Materials

The following supporting information can be downloaded at https://www.mdpi.com/article/10.3390/biom14070821/s1, Figure S1: Activating ligand of excitatory DREADD vector, clozapine-N-oxide (CNO), does not significantly alter skeletal muscle temperature in wild-type control mice, Figure S2: SF1-Cre+ mice with mCherry control vector were not significantly altered by clozapine-N-oxide (CNO) or predator odor, Figure S3: Metabolic parameters not significantly altered in SF1-Cre+ mice with mCherry control vector, and Figure S4: Metabolic parameters not significantly altered in SF1-Cre+ mice with mCherry control vector during controlled activity. Table S1: The dataset before compared to after the application of the inclusion criterion. The application of the inclusion criterion did not alter the determination of significance.

## Abbreviations

Anterior hypothalamic nucleus (AHN); area under the curve (AUC); central/dorsomedial ventromedial hypothalamus (c/dmVMH); clozapine N-oxide (CNO); designer receptors exclusively activated by designer drugs (DREADD); energy expenditure (EE); periaqueductal gray (PAG); predator odor (PO); respiratory exchange ratio (RER); steroidogenic factor 1 (SF1); vehicle (Veh); ventromedial hypothalamus (VMH); oxygen consumption (VO_2_); wild-type mouse (WT).
