# Supplementary material for: Chemogenetic Excitation of Ventromedial Hypothalamic Steroidogenic Factor 1 (SF1) Neurons Increases Muscle Thermogenesis in Mice
Source: Biomolecules. 2024 Jul 9;14(7):821. doi: 10.3390/biom14070821 (PMC11274921; doi:10.3390/biom14070821)
Supplement: Supplementary file 1 [file biomolecules-14-00821-s001.zip › biomolecules-3019951-supplementary.pdf]

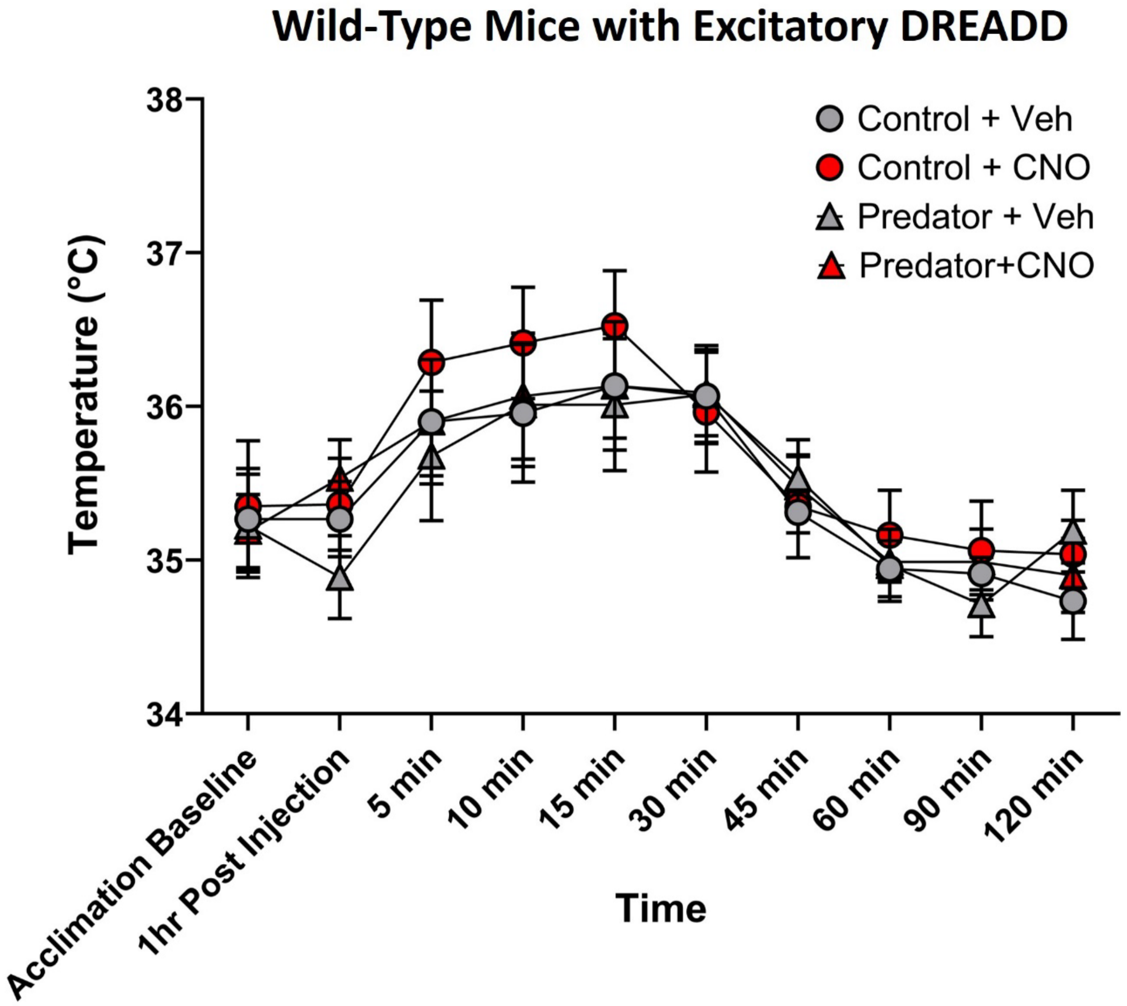

Figure S1: Activating ligand of excitatory DREADD vector, clozapine-N-oxide (CNO), does not significantly alter skeletal muscle temperature in wild-type control mice. Gastrocnemius muscle temperature of wild-type mice transduced pAAV-hSyn-DIO-hM3D(Gq)-mCherry (AAV8) following CNO or vehicle (1mg/kg, i.p.). Thermogenesis was measured in all mice in each condition. N = 8, error bars represent ±SEM. PO, predator odor; Control, control odor; CNO, clozapine-N-oxide; Veh, vehicle (sterile saline).

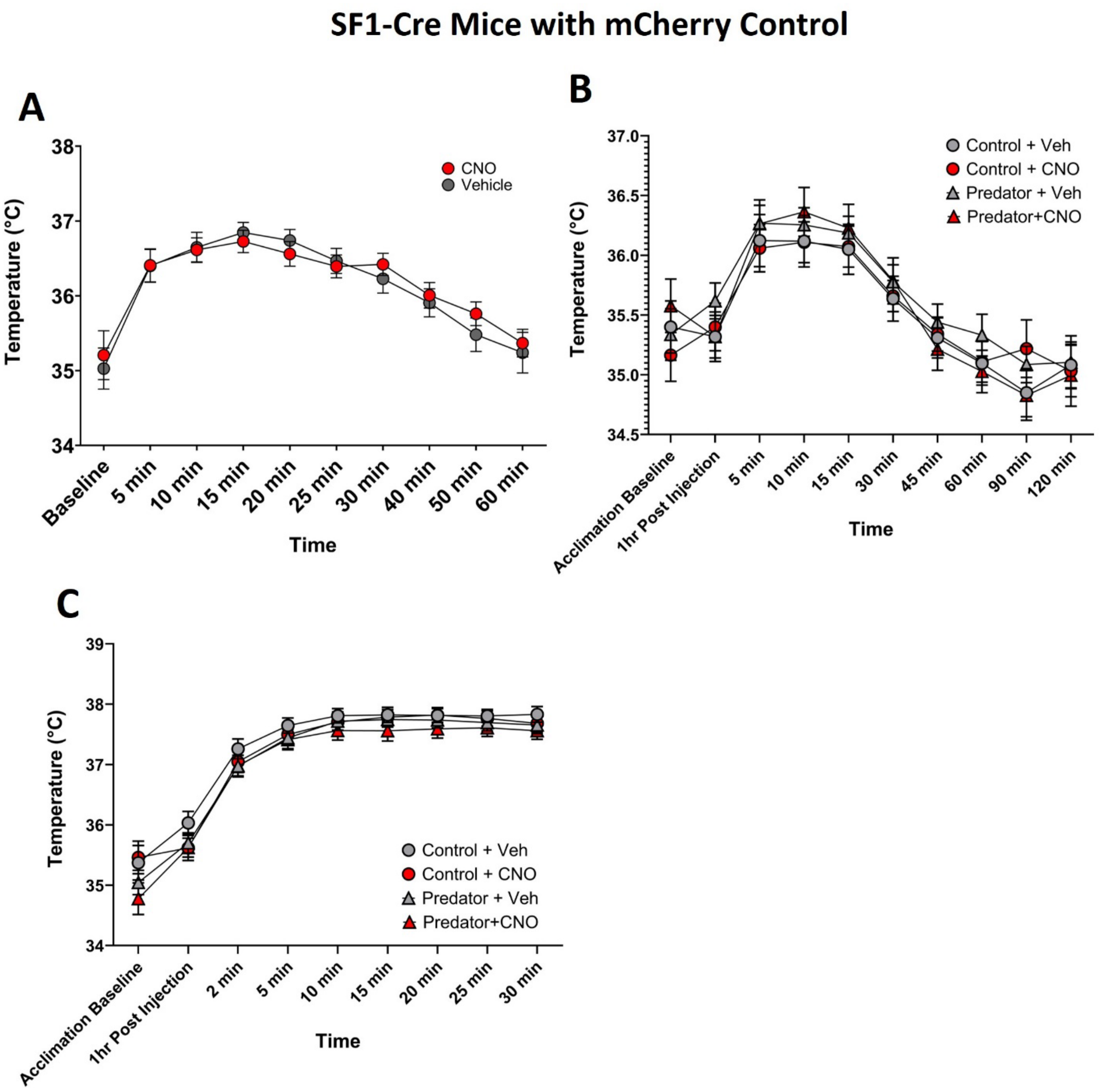

Figure S2: SF1-Cre+ mice with mCherry control vector were not significantly altered by clozapine-N-oxide (CNO) or predator odor. Gastrocnemius muscle temperature of SF1-Cre+ mice transduced pAAV-hSyn-DIO-mCherry (AAV8), control virus following CNO or vehicle (1mg/kg, i.p.) in (A) home-cage or (B) controlled activity settings. Thermogenesis was measured in all mice in each condition. N = 15, error bars represent ±SEM. PO, predator odor; Control, control odor; CNO, clozapine-N-oxide; Veh, vehicle (sterile saline).

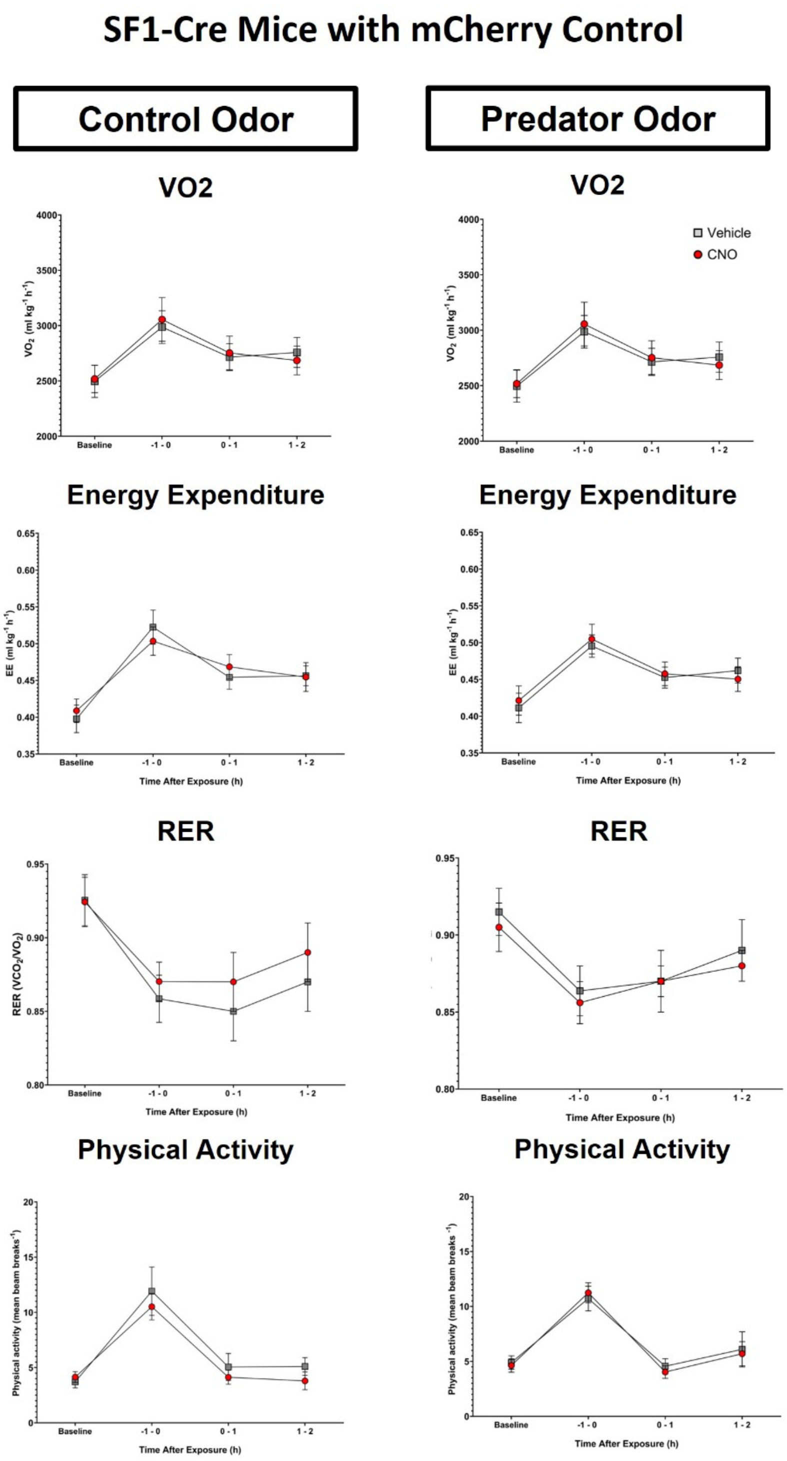

Figure S3: Metabolic parameters not significantly altered in SF1-Cre+ mice with mCherry control vector. Oxygen consumption (VO2), energy expenditure (EE), respiratory exchange ratio (RER; VCO_2/VO_2), and physical activity of SF1-Cre+ mice transduced pAAV-hSyn-DIO-mCherry (AAV8), control virus following CNO or vehicle (1mg/kg, i.p.) and presented with **[left]** control odor and **[right]** predator odor. All mice were tested in each condition. N = 15, $*p < 0.05$, error bars represent ±SEM. CNO, clozapine-N-oxide; Vehicle, sterile saline.

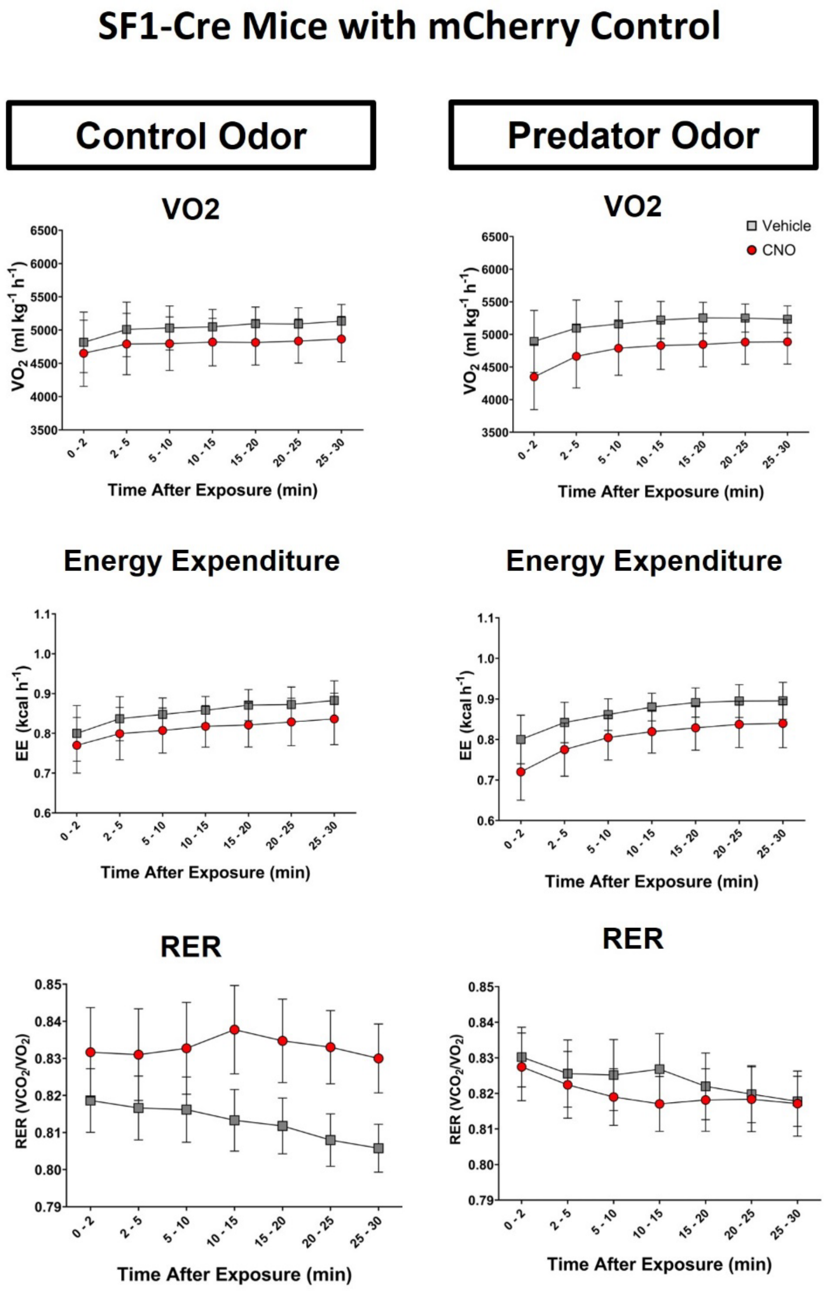

Figure S4: Metabolic parameters not significantly altered in SF1-Cre+ mice with mCherry control vector during controlled activity. Oxygen consumption (VO_2), energy expenditure (EE), respiratory exchange ratio (RER; VCO_2/VO_2), and physical activity of SF1-Cre+ mice mice transduced pAAV-hSyn-DIO-mCherry (AAV8), control virus following CNO or vehicle (1mg/kg, i.p.) and presented with control odor **[left]** and predator odor **[right]**. All mice were tested in each condition. Mice were consecutively measured for 30 min, one hour following injection. N = 14, error bars represent ±SEM. CNO, clozapine-N-oxide; Vehicle, sterile saline.

Table S1: Dataset before compared to after application of inclusion criterion. The application of the inclusion criterion did not alter the determination of significance.

**Dependent Variable**	**Comparison**	**N**	**Significance (*p*-value)**	**Different from inclusion criteria?**
Muscle Temperature (°C)	Time<br>Vehicle v CNO<br>Control v PO	16	<0.001<br>0.002<br>*n.s.*	No
Muscle Temperature (°C) *controlled activity*	Time<br>Vehicle v CNO<br>Control v PO	16	<0.001<br>0.001<br>*n.s.*	No<br>Yes, p = 0.02<br>No
VO_2 (ml/kg/hr)	Time<br>Vehicle v CNO<br>Control v PO	16	<0.001<br>0.007<br>*n.s.*	No<br>Yes, p = 0.02<br>No
EE (kcal/hr)	Time<br>Vehicle v CNO<br>Control v PO	16	<0.001<br>0.02<br>*n.s.*	No<br>Yes, p = *n.s.*<br>No
RER (VCO_2/VO_2)	Time<br>Vehicle v CNO<br>Control v PO	16	<0.001<br>*n.s.*<br>*n.s.*	No
Activity (beam breaks/min)	Time<br>Vehicle v CNO<br>Control v PO	16	<0.001<br>*n.s.*<br>*n.s.*	No
VO_2 (ml/kg/hr) *controlled activity*	Time<br>Vehicle v CNO<br>Control v PO	15	*n.s.*<br>*n.s.*<br>*n.s.*	No
EE (kcal/hr) *controlled activity*	Time<br>Vehicle v CNO<br>Control v PO	15	*n.s.*<br>*n.s.*<br>*n.s.*	No
RER (VCO_2/VO_2) *controlled activity*	Time<br>Vehicle v CNO<br>Control v PO	15	*n.s.*<br>*n.s.*<br>*n.s.*	No

CNO, clozapine-N-oxide; PO, predator odor; VO_2*, oxygen consumption; EE, energy expenditure; RER, respiratory exchange ratio*
